# Nutritional Profile, Disease Severity, and Quality of Life of Patients with Inflammatory Bowel Disease: A Case–Control Study

**DOI:** 10.3390/nu16121826

**Published:** 2024-06-11

**Authors:** Lea N. Sayegh, Firas Haddad, Layane Bou Jaoude, Nicole Fakhoury-Sayegh, Gessica N. H. A. Heraoui, Zainab Nasrallah, Charbel Chidiac, Rashad Nawfal, Fadi F. Francis, Fadi H. Mourad, Jana G. Hashash

**Affiliations:** 1 Department of Gastroenterology and Hepatology, Mayo Clinic, 200 1st St. SW, Rochester, MN 55905, USA; lrs08@mail.aub.edu; 2 School of Medicine, American University of Beirut, Bliss St., Beirut P.O. Box 11-0236, Lebanon; ffh07@mail.aub.edu (F.H.); lrb12@mail.aub.edu (L.B.J.); zanasra@iu.edu (Z.N.); cchidia2@jh.edu (C.C.); 3 Department of Nutrition, Saint Joseph University, Damascus St., Beirut P.O. Box 17-5208, Lebanon; nicolsayegh@gmail.com (N.F.-S.); h.gessica@gmail.com (G.N.H.A.H.); 4 Department of Internal Medicine, Indiana University, 1120 W Michigan St., Indianapolis, IN 46202, USA; 5 Department of Surgery, Johns Hopkins School of Medicine, 1800 Orleans St., Baltimore, MD 21287, USA; 6 Department of Anatomy, Cell Biology and Physiological Sciences, American University of Beirut, Bliss Street, Beirut P.O. Box 11-0236, Lebanon; rashad_nawfal@dfci.harvard.edu; 7 Department of Medical Oncology, Dana Farber Cancer Institute, 450 Brookline Ave., Boston, MA 02215, USA; 8 Division of Gastroenterology, Hepatology, and Nutrition, University of Pittsburgh, 200 Lothrop St., Pittsburgh, PA 15261, USA; francisff2@upmc.edu; 9 Department of Gastroenterology and Hepatology, American University of Beirut Medical Center, Cairo Street, Beirut P.O. Box 11-0236, Lebanon; fmourad@aub.edu.lb; 10 Division of Gastroenterology and Hepatology, Mayo Clinic, Jacksonville, FL 32224, USA

**Keywords:** IBD, diet, quality of life, nutritional profile

## Abstract

Introduction: Diet is thought to play an important role in the clinical course and quality of life (QOL) of patients with inflammatory bowel disease (IBD). However, dietary habits of patients with IBD are still unknown. This case–control study aims to compare the dietary habits of patients with IBD to healthy controls and evaluate differences in disease severity and QOL. Materials and methods: Food frequency, severity scores using the Harvey–Bradshaw and Ulcerative colitis activity index, and QOL were assessed using online questionnaires. Dietary habits were compared for patients with active disease and remission and for those with low QOL (LQOL) and high QOL (HQOL). Results: We recruited 61 patients with IBD and 101 controls. Significance was set at *p* = 0.05. Controls consumed significantly more daily calories (2546 vs. 1641, *p* = 0.001). However, patients with IBD consumed a higher percentage of carbohydrates (50% vs. 45%, *p* = 0.001), more red meat (*p* = 0.024), and less fiber, sucrose, and lactose (*p* = 0.001, 0.001, and 0.036). Patients with active disease had higher lipid intake, lower protein intake, and lower QOL (47 vs. 58, *p* = 0.001). Dietary differences between LQOL and HQOL mirrored those between active disease and remission. Conclusion: This study is the first to provide valuable insights into the nutritional profile of Lebanese patients with IBD.

## 1. Introduction

Inflammatory bowel diseases (IBD) encompass two primary conditions: ulcerative colitis (UC) and Crohn’s disease (CD) [1]. The prevalence of these chronic conditions has shown a consistent increase over time [2,3]. The incidence of UC is estimated to be 6.3 per 100,000 person-years in Asia and the Middle East, and that of CD 12.7 per 100,000 person-years [2]. It is projected that the prevalence of IBD is expected to increase by 2.3 times from 2020 to 2035, suggesting the need to better understand this disease in this specific population [4]. While the precise pathophysiology of IBD remains elusive, it is widely recognized to involve a complex interplay between genetic predisposition and environmental influences [5]. The investigation of environmental factors holds significance as these represent modifiable risk factors for IBD, offering potential avenues to alleviate the global burden of the disease and enhance the quality of life (QOL) of affected individuals.

Diet stands out as a pivotal factor of interest in IBD. However, its precise role in disease onset, severity, and progression remains incompletely understood [6]. Despite the ESPEN guidelines on Clinical Nutrition for patients with IBD, confusion and uncertainty amongst patients and physicians persist [7,8,9]. The absence of standardized dietary recommendations often prompts patients with IBD to adopt self-identified dietary patterns. For instance, despite the absence of official endorsements, approximately 70% of patients resort to elimination diets during exacerbation periods, which can lead to malnutrition and significantly compromise their quality of life [6]. Presently, patients with IBD are advised to pursue diets rich in essential vitamins and nutrients, with particular emphasis on vitamin D, since vitamin D deficiency has been linked to disease severity [10,11,12]. Studies have also investigated the potential benefits of omega-3 fatty acid and probiotic supplementation in mitigating disease severity and progression [13]. Additionally, certain dietary regimens such as the low fermentable oligosaccharides, disaccharides, monosaccharides, and polyols (FODMAP) diet have been explored for their potential efficacy in managing IBD [14]. Notably, research indicates that diets high in saturated fats, monosaccharides, and sugar-sweetened beverages may contribute to adverse outcomes in patients with IBD [15,16,17]. On the other hand, a study evaluating diet in patients newly diagnosed with IBD showed that a high intake of fruits and vegetables was associated with a decreased onset of UC and CD [18].

When evaluating what patients with IBD actually eat, a European study found that patients with CD consumed significantly lower amounts of fiber and vitamins than healthy subjects and had lower concentrations of total cholesterol and amino acids [19]. In addition, a study conducted in Jordan found a significantly higher degree of malnutrition among patients with IBD when compared to controls, as well as lower BMI and waist circumference. Patients with IBD were also much more likely to skip a meal [20].

The Lebanese diet is known to be rich in fruits and vegetables; however, the general improvement in standard of living in the early 2000s has shifted the trend to a more “westernized” type of diet, including high-fat and high-salt processed food [21]. Thus, describing the diet of Lebanese patients with IBD could shed light on the dietary habit of this specific group in the context of evolving dietary trends in Lebanon.

Given the heterogeneous nature of data investigating the association between diet and IBD, and the lack of knowledge about dietary habits among Lebanese patients with IBD, the objective of this study is to conduct a case–control investigation evaluating the nutritional profile of patients with IBD and compare it to age-matched controls without IBD. As a secondary outcome, the nutritional profile and quality of life of patients with active disease will be compared to the profile of patients in remission.

## 2. Materials and Methods

### 2.1. Study Design and Sampling Strategy

This is a case–control study which was conducted over a period of 30 months, from September 2021 to February 2024. Cases were identified through review of the electronic medical records and then contacted via telephone. Eligible participants were patients with IBD aged 18–75 years who live in Lebanon and have a medical file at our institution. Once patients were screened for inclusion and exclusion criteria, and they consented to participate in the study, they were asked to fill out the Food Frequency Questionnaire (FFQ), IBD severity scores, and IBD-related QOL questionnaires. Inclusion criteria included any patient between the ages of 18 and 75 years who was formally diagnosed with either UC or CD by one of the gastroenterologists at our institution, and who had the diagnosis added to their problem list in their chart. Patients were excluded if they had an indeterminate diagnosis, were below the age of 18 or above age 75 years, and did not have at least one visit with a gastroenterologist at our institution.

For recruitment of controls, emails were sent to faculty members, staff, and the student body at the American University of Beirut and its affiliated hospital the American University of Beirut Medical Center. Controls were included if they were between the ages of 18 and 75 years, lived in Lebanon, and did not have IBD. These participants only filled out the general demographics and FFQ questionnaires.

### 2.2. Measurement Techniques

1. The sociodemographic status of participants and their anthropometric measures, exercise, social habits, and general symptoms were obtained via a questionnaire. This included a total of 28 questions.
2. The FFQ was available in English and in Arabic, and both versions were previously validated for the typical Lebanese diet [22]. The FFQ is divided into 12 food groups that represent the overall intake of the previous year. The frequency is divided into 8 distinct categories to facilitate analysis. The reported frequencies were converted to a daily equivalent to standardize the data. For instance, if a participant reported eating a particular food item 3 times per week, this frequency was extrapolated to a daily rate by dividing by 7 (i.e., 3/7 times per day). Macronutrient and micronutrient composition were then extracted from each food item and summed up for each participant.
3. IBD-related quality of life was evaluated by the short-IBD questionnaire (SIBDQ) [23,24], which is also validated in an Arabic version [25].
4. The severity of CD was assessed by the Harvey–Bradshaw CD questionnaire (HBCD) [26] and UC by the UC activity index (UCAI) [27]. Additional questions were asked about flares and hospitalizations during the past year as surrogates for disease severity over that period.

### 2.3. Data Collection Procedures and Plan of Analysis

After patients consented to participate in the study, they answered the questionnaires in the language they prefer (English or Arabic), either through an online survey or via telephone. Controls answered questions about their demographics and the FFQ only.

According to the HBCD questionnaire results, patients with CD were divided into three categories: (1) patients with a score of 4 or less who were considered to be in clinical remission, (2) patients with a score of 5 to 6 who were considered to have mild-to-moderate disease, and [24] patients with a score of 7 or more who were considered to have severe disease. Using UCAI results, patients with UC were also divided into three groups: (1) patients with index scores of 2 or less who were considered to be in clinical remission, (2) patients with scores of 3 to 5 who were considered to have mild-to-moderate disease, and Ref. [24] patients with a score above 5 who were considered to have severe disease. Since the sample size and individual groups are small, the mild, moderate, and severe groups were clustered together for analysis into a “clinically active disease subgroup” for both UC and CD patients, while those who were considered in clinical remission in both the UC and CD groups were clustered into a “remission” group.

As for the SIBDQ, the scores range from 1 to 7 for each question, with 1 being a very poor quality of life and 7 being a very good quality of life [28]. Participants were categorized into two groups: (1) those with a score less than 60 as having a low QOL (LQOL), or (2) those with a score of 60 or more as having a high QOL (HQOL) [29]. We performed an independent *t*-test with the QOL score as the dependent variable and clinical severity scores (HBCD and UCAI) as the independent variable, to assess correlation of QOL with clinical severity of disease.

The data from the FFQ were logged into Nutrilog (Nutrilog, Marans, France, version 2.33) [30] to evaluate for calories, macronutrients, and micronutrients. We compared the amounts and mean percentages of daily caloric intake of different macro- and micronutrients, as well as alcohol consumption between cases and controls and between cases with clinically active disease versus those in clinical remission. We also compared demographic and anthropometric parameters between cases and controls, to account for differences between the two groups. Among those parameters, crowding index was used as a surrogate measurement of socioeconomic status, as the unemployment category contained a large number of students, owing to the nature of data collection among controls. A crowding index > 1 was considered a low socioeconomic status [31]. Physical activity was also categorized as physically active or inactive, using the WHO classification [32].

### 2.4. Statistical Analyses

In sum, continuous variables were expressed as means and standard deviation, and comparison between variables was performed using an independent *t*-test when normally distributed, while median with interquartile range (IQR) was used for variables that are not normally distributed, and, in this case, comparison was carried out using Mann–Whitney U testing. As an additional descriptive table, we also compared patients with UC and CD in terms of number of hospitalizations, flares, diet, and medication change to assess severity over the previous year. The associations between proportions and percentages were analyzed using Chi-square test, and the Fisher exact test was used when expected counts were less than 5. All tests are two-tailed, and the significance level was set at *p* < 0.05, with a confidence interval of 95%. The statistical analysis was carried out using SPSS 25 for Windows (IBM Corp., Released 2011, IBM SPSS Statistics for Windows, Version 25.0, Armonk, NY, USA).

## 3. Results

### 3.1. Patient Characteristics

A total of 61 patients with IBD (31 UC and 30 CD) and 101 controls were enrolled. Amongst the patients with IBD, 47% were males and 53% females, with a mean age of 40 ± 14 years (mean ± SD). The median numbers of years between date of diagnosis and filling out the questionnaire was found to be 2 (1–7) and ranged from 0.5 years to 31 years. As for the controls, 39% were males and 61% females, and their mean age was 36 ± 17 years. On average, 81.4% of cases had a university degree compared to 91.1% of controls. A third of the patients with IBD (36%) had a high crowding index > 1, while 21.1% of controls had a high crowding index; however, the difference was not statistically significant. There was no significant difference between cases and controls in terms of personal medical history or family medical history.

Regarding sociodemographic, anthropometric, and environmental parameters, most of these revealed no significant differences between cases and controls except for smoking. The percentage of smokers was significantly higher in cases than in controls (24.6% vs. 10.9%, *p* = 0.021). All patients’ characteristics are presented in Table 1.

### 3.2. Severity Scores and Quality of Life

Of 61 patients with IBD, only 47 filled out the severity score and QOL questionnaires (25 patients with UC and 22 patients with CD). For patients with UC, 11 (44%) had a UCAI score ≤ 2, indicating clinical remission of disease, while 8 (32%) had a score between 3 and 5, indicating mild-to-moderate disease, and 6 (24%) had a score of 6 or more, indicating severe disease. Of 22 patients with CD, 17 (77.3%) had a score of 0 to 4, indicating clinical remission, 3 (13.6%) had a score of 5 to 6 and thus had mild-to-moderate disease, while 2 (9.1%) had a score of 7 or higher, indicating severe disease.

**Figure 1:**
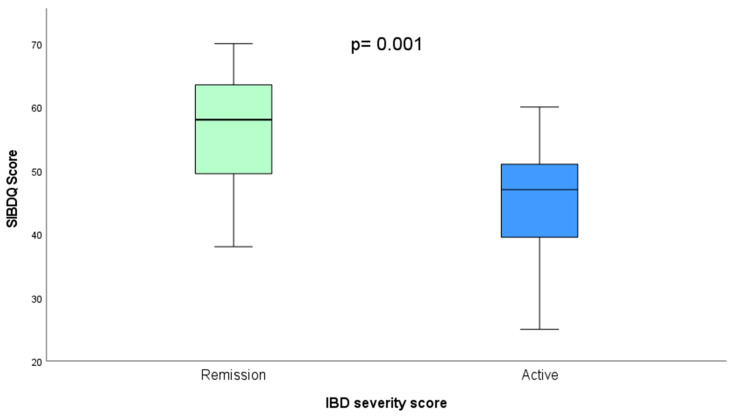
Boxplot comparing median values for quality of life (QOL) scores between patients in clinical remission and those with active disease.

The mean score for QOL was equal between patients with UC and CD at 51.64. Thirty-three patients were classified as having a LQOL (score < 60) and 14 as having a HQOL (score ≥ 60). The median QOL was significantly higher in patients with IBD in clinical remission compared to patients with active disease (58 vs. 47, *p* = 0.001) (Figure 1).

The majority of patients (68%) reported having at least 1 flare up of their IBD disease activity in the past year, with 62% reporting changing their diet due to the flare. A quarter of patients (26%) have been hospitalized and/or had surgery in the previous year, and 34% had to change their IBD-related medication (Table 2).

### 3.3. Dietary Data

#### 3.3.1. Cases vs. Controls

Median daily energy intake (kcals/day) was significantly different between cases and controls, with controls consuming more daily calories (2546 vs. 1641, *p* = 0.001). For males, cases consumed 1609 kcals/day, while controls consumed 2588 kcals/day (*p* = 0.001). For females, cases consumed 1656 kcals/day, while controls consumed 2507 kcals/day (Table 3).

Controls also consumed a significantly higher amount of all macronutrients per day, including protein (99.5 g vs. 77.5 g, *p* = 0.001), lipids (37.6 g vs. 31.5 g, *p* = 0.001), and carbohydrates (295 g vs. 217 g, *p* = 0.001). This was also the case with lipid subtypes, such as mono-unsaturated fatty acids (MUFAs), poly-unsaturated fatty acids (PUFAs), and saturated fatty acids (SFAs) (Table 3).

**Figure 2:**
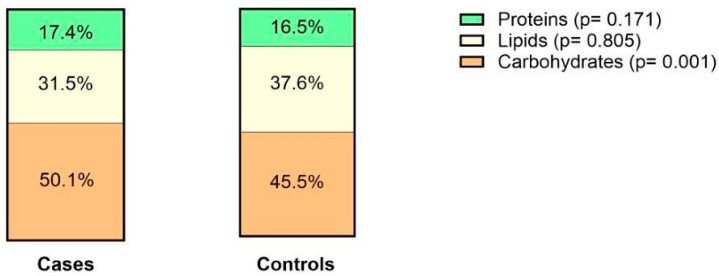
Average percentage of different macronutrient intake per daily caloric intake in cases and controls.

When looking at the composition of the daily food intake, the percentage of protein consumption was not different between the two groups (*p* = 0.17). In contrast, cases consumed a higher percentage of carbohydrates than controls (50.1% vs. 45.5%, *p* = 0.001). The percentage of lipids was not significantly different between cases and controls (31.5% vs. 37.6%) (Figure 2). When analyzing lipid subtypes, the percentage of SFAs (11.7 vs. 31.8) consumption was significantly different between both groups (*p* = 0.001); however, percent MUFAs and PUFAs were not significantly different (16.1 vs. 32.4, *p* = 0.07 and 8.9 vs. 16.3, *p* = 0.97). In addition, there was no significant difference in consumption of dairy, vegetables, or fruits between cases and controls; however, cases ate significantly more red meat than their healthy counterparts (Table 3). Cases consumed a significantly lower amount of fiber, lactose, and sucrose than controls. Alcohol consumption was not different between groups (Table 3).

#### 3.3.2. Patients in Clinical Remission vs. Those in Active Disease

Median energy intake was not significantly different between patients in remission and those with active disease, regardless of gender (Supplementary Table S1).

Consumption of total amount of proteins, carbohydrates, and lipids was similar between both groups. Lipid subtypes consumption of MUFAs and SFAs were also similar; however, patients with active disease consumed a significantly larger amount of PUFAs (11.7 g vs. 5.6 g, *p* = 0.041).

**Figure 3:**
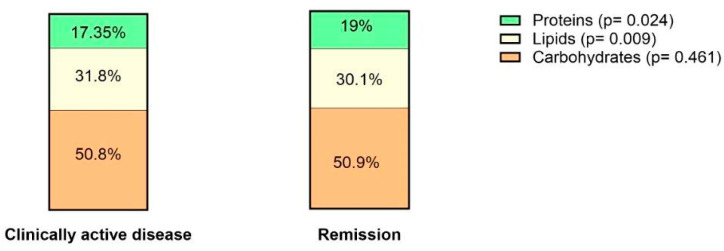
Median percentage of different macronutrient intake per daily caloric intake in patients with clinically active disease versus those in remission.

When looking at the composition of the consumed diet, the average percentage of proteins was significantly higher in the remission group, while the carbohydrates percentage was similar between both groups (Figure 3). The average percentage of lipids of total energy intake was significantly higher in patients with active disease. When looking at lipid subtypes, patients with active disease consumed a significantly higher percentage of PUFAs, specifically omega-6 fatty acids as compared to those in remission (6.45 g vs. 5.00 g, *p* = 0.012), while percentages of MUFAs and SFAs were similar. In contrast, for all other evaluated nutrients and alcohol, there was no significant difference between both groups (Supplementary Table S1).

As for food groups, patients with active disease consumed a significantly lower amount of fruits than those in remission (*p* = 0.03). All other food groups evaluated were not significantly different between both groups (Supplementary Table S1).

#### 3.3.3. Patients with LQOL vs. Patients with HQOL

Median daily energy intake was significantly different between the LQOL and HQOL groups (1688 vs. 1517, *p* = 0.003); however, this difference was not present when looking at caloric intake among men (Supplementary Table S2).

Daily consumption of proteins and lipids was higher among the LQOL group, while consumption of carbohydrates was similar between groups. When looking at lipid subtypes, the LQOL group consumed a significantly higher amount of PUFAs (11.7 g vs. 8.2 g) and SFAs (7.2 g vs. 5.9 g) than the HQOL group. Consumption of MUFAs was similar between both groups.

**Figure 4:**
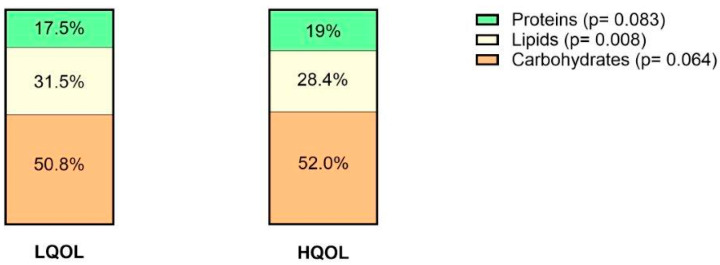
Median percentage of different macronutrient intake per daily caloric intake in patients with LQOL versus those with HQOL.

When looking at percent consumption of daily intake, the LQOL group was found to consume a higher percentage of lipids, and similar percentages of carbohydrates and proteins (Figure 4). Patients with LQOL consumed a significantly higher percentage of PUFAs (6.0 vs. 4.8) and SFAs (7.6 vs. 6.0). There was no difference when looking at omega-3 and omega-6 subtypes. The percentage consumption of MUFAs was also similar. Those with LQOL also consumed higher amounts of sucrose (51 g vs. 37.1 g). All other evaluated nutrients and alcohol were not statistically significant between both groups (Supplementary Table S2).

## 4. Discussion

This study compares the nutritional profiles of 61 Lebanese patients with IBD and 101 healthy controls. Our study found many differences in the nutritional profile of these two groups, specifically total calorie consumption, percent macronutrients of daily caloric intake, as well as fiber, sucrose, and lactose. Patients with IBD consumed significantly fewer calories and, by extension, a lower total amount of protein, fat, and carbohydrates. However, when looking at percent of total caloric intake, percent protein and lipids were similar, while patients with IBD consumed higher percent carbohydrates. They also consumed a lower percent of SFAs compared to controls, and less total amount of sucrose, lactose, and fiber. When looking at food groups, they consumed a smaller amount of red meat and similar amounts of dairy, vegetables, and fruits. In Lebanon, the Mediterranean diet used to be highly prevalent; however, modernization and improvement in standard of living have led to an increase in adherence to a more “Westernized diet” [21]. Compared to healthy counterparts, patients with IBD seem to be switching back to the former type of diet, which is typically lower in saturated fats and in refined sugars such as sucrose, although typically higher in fiber content.

Baseline characteristics between the cases and controls are mostly similar, except for smoking. The effects of smoking tobacco on incidence of IBD and subsequent disease course differ, with protective effects in UC and worse effects in CD [34]. The higher prevalence of smokers among the UC group could be because of learned behavior and associated symptom relief or could be the result of random sampling error. Since there is a similar percentage of smokers in the CD group, this might favor the latter hypothesis.

As previously mentioned, in our cohort, patients with IBD consumed a significantly lower amount of total calories and, by extension, a lower amount of proteins, lipids, and carbohydrates than their healthy counterparts. This is in accordance with a study conducted in Jordan that found that patients with IBD had a significantly lower BMI and waist circumference than healthy subjects, were more likely to skip a meal, and had higher degrees of malnourishment [20]. This discrepancy might also be explained by the anorexia that patients with IBD may experience, especially during periods of disease activity, or from fear of eating certain foods to avoid disease flares, as stated above [35]. Interestingly, 61.7% of patients in our cohort reported changing their diet due to flares. The exact reasons for these changes are unknown, as no specific dietary recommendations are given to patients in our clinics, owing to scarce available information in our region. Patients might be attempting to change their diets based on personal beliefs or trial and error. In contrast, percent consumption of different macronutrients differed between both groups only with respect to carbohydrates, with a higher percentage of carbohydrates among cases (50 vs. 45), while percentage of protein (17.4 vs. 16.5) and percentage of lipids (31.5 vs. 37.6) were similar.

Studies on dietary fat and the risk of developing IBD are mixed. Some retrospective studies suggest an increased risk of UC and CD with high total fat intake; however, a few prospective studies showed no association [3,36]. Mixed results were also seen when evaluating PUFAs, MUFAs, and SFAs, and many studies reported no significant difference in consumption between the patients with IBD and healthy controls [3,36]. In contrast, cases in our cohort consumed significantly less SFAs, MUFAs, and PUFAs than controls in terms of total amount and less SFAs in terms of percentage of daily caloric intake. This might again be due to voluntary dietary changes patients have made to manage their disease. Interestingly, when comparing lipid consumption between cases with active disease and those in remission, there was no significant difference in total lipid consumption; however, the former group consumes significantly more percent lipids, as well as total and percent PUFAs, specifically omega-6 fatty acids. Several studies suggested that an imbalance in *n*-6/*n*-3 (Omega 6 to Omega 3) may contribute to a pro-inflammatory state that might worsen disease severity in patients with IBD [37,38], which is mirrored in our results.

As for carbohydrates, although patients with IBD consumed a significantly smaller total amount of carbohydrates per day, they consumed a significantly higher percentage of carbohydrates than controls. Studies have not shown any significant association between carbohydrates and incidence of IBD [3,36]; however, there is evidence that a higher amount of refined carbohydrates such as sucrose can be related to IBD onset [3]. Interestingly, cases in our cohort consumed a significantly smaller amount of sucrose than controls. This might be in part due to conscious changes in their dietary habits and avoidance of high-fat food groups. Patients might prefer filling meals that are low in fiber and fat content such as potatoes, pasta, or rice. Conversely, patients with active disease consumed more sucrose, which is a refined carbohydrate, than those in remission, which is consistent with the idea that refined sugars may be associated with relapse [17]. Preclinical studies have also looked at this relationship, and mice placed on a high-sugar diet had increased susceptibility to colitis and an increase in concentration of pro-inflammatory cytokines [39,40,41].

As for proteins, cases consumed a smaller total amount of daily proteins, but there was no significant difference in percent consumption between cases and controls. Data on the effect of protein intake on IBD are mixed, but a large prospective study of more than 60,000 participants has shown a positive association between protein intake and onset of IBD [3,42]. However, in our sample, patients with IBD in remission were found to consume a significantly higher percentage of protein than those with active disease, while no difference was found in the total amount of consumed protein between both groups, suggesting that a higher proportion of protein in the diet might be associated with a lower rate of relapse, possibly due to the lower resulting percentage of fat consumption. According to the 2023 American Gastroenterology Association (AGA) guidelines, a diet such as the Mediterranean diet, which is higher in lean protein and complex carbohydrates and low in fats (consisting mostly of MUFAs), while limiting red meat consumption, is advised for patients with IBD [43].

Interestingly, when evaluating meats, dairy, vegetables, and fruits, only red meat consumption was found to be significantly different between both groups. A review by Khalili et al. also suggests that high red meat consumption is associated with an increased risk of IBD [44]. Between patients in remission and those with active disease, there is only a significant difference between both groups in terms of fruit intake, which is higher for patients in remission. This is in accordance with previous studies, whereby fruits are strongly recommended for maintenance of remission in patients with IBD [42]. Vegetables and dairy are also encouraged, while meats, especially red meats are thought to provoke relapse and should be avoided, as mentioned previously [42].

Fiber intake per day is much lower in the patients with IBD than in the control group. This is similar to what was observed in previous studies that showed that patients with IBD tend to avoid fiber, eating less fiber per 1000 kcal than controls [36]. Anecdotally, many patients reported actively avoiding fiber as they believe fiber is associated with their disease flares and reported increased relief when they adhered to a low-fiber diet. However, it was suggested that although many patients benefit in the short term from reduction of fiber intake, these restrictions can lower fecal microbiota abundance and decrease essential nutrient intake [45]. Changes in microbiota have also been shown to impact IBD progression [46] and development of colorectal cancer [47], a feared complication of chronic IBD. Thus, recommending adequate fiber intake might be crucial in this patient population. There is also evidence that patients can tolerate introduction of fiber if it is done during periods of remission [45]. Current guidelines are to avoid restrictions of fiber in diet, although the benefit of supplementation in the long term is unknown [45]. Another study found that limiting fiber increased the risk of a flare in patients with CD but had no effect on patients with UC [48]. In addition, fiber consumption fell short of the recommended daily intake of at least 25 g for women and 31 g for men [49]. Interestingly, no significant difference was found between fiber intake of cases of remission versus those with active disease, suggesting no relation between fiber intake and disease activity.

Studies have shown an association between alcohol and relapse in patients with UC [50]. Patients with inactive IBD also report worsening of their disease with alcohol consumption [51]. In our sample, there was no observed difference between cases and controls or between those with active disease and those in remission in terms of alcohol consumption. However, the absolute number of patients who consumed alcohol in the IBD group was low (six with active disease and four in remission), so any significant association might not be apparent without a larger sample size.

As expected, QOL was significantly lower in patients with active disease as compared to those in remission. As such, evaluation of dietary components of patients in remission can clue us in to dietary habits of patients with IBD with a better overall QOL. When comparing the nutritional profile of LQOL vs. HQOL, those with a better overall QOL consumed less lipids, PUFAs, SFAs in absolute values and in percentages, and sucrose than their counterparts, which correlates with the results found in those between remission and active disease. They also consumed fewer calories and less total protein; however, the percentage protein was not different between both groups, and the smaller total amount might be due to fewer overall calories.

As stated above, many of the discrepancies found in our current study might be explained by the method of data collection. Since patients who have been interviewed have been diagnosed and been under treatment for many years, they might have adjusted their diet based on their perceived knowledge of which food groups might cause their disease to flare. It could account for decreased fat and fiber intake as well. In addition, the study is not powered to detect differences in food consumption between patients with IBD in remission and those with active disease; thus, these differences might be missed due to small sample size.

There are many limitations to our current study. There is an inherent selection bias within the control group, as participants were recruited via email from university staff and students. Other sources of bias represent social bias and recall bias, as patients might under- or overestimate the proportions of certain food items, while overestimating the amount of exercise they do. They might also simply not remember certain details. In addition, many patients had been diagnosed with IBD for many years prior to administration of the questionnaire. Therefore, we cannot infer a link between dietary habits and IBD incidence; however, the present study does provide insight into general dietary habits of patients with IBD in Lebanon and may provide insight as to which food groups patients might be avoiding, as well as dietary differences between those with active disease and poor QOL with those in remission with a better QOL. Some associations that might have been statistically significant might also have been missed due to the small sample size, particularly in the analysis of active versus quiescent disease. Recruitment was also made difficult by the surrounding economic crisis, and many patients were unwilling to contribute their time to answer the lengthy questionnaire.

To our knowledge, this is the first study to examine the dietary habits of patients with IBD in Lebanon and compare it to healthy controls. Although we cannot infer a relationship between diet and incidence of IBD, we were able to describe differences between dietary habits of patients with IBD and their healthy counterparts. Future prospective studies are needed to evaluate pre-illness diet with incidence of IBD, as well longitudinal follow-up of patients for disease flares with rigorous follow-up of dietary habits. Many patients reported changing their diet because of their disease or during a flare and would benefit from specific recommendations and rigorous nutritional education.

## 5. Conclusions

In conclusion, the present study describes the dietary habits of Lebanese patients with IBD and displays the difference with the diet of healthy subjects, with the former seemingly eating less saturated fatty acids and fewer total calories, as well as avoiding fiber, with many admitting to changing their diets due to flares. Our study also elucidates the need for evaluation of dietary habits and their association with IBD onset and disease course and can provide groundwork for evaluation of these relationships in Lebanese patients with IBD specifically. Currently, we can advise that patients follow a nutritionally complete diet, correcting nutritional deficiencies, as well as attempting to adopt a diet like the Mediterranean diet, namely rich in complex carbohydrates, MUFAs, fiber, lean protein, fruits, and vegetables. It might also be beneficial to reach the daily required intake for fruits and fiber and counsel patients not to avoid them.

## Figures and Tables

**Table 1:** Baseline sociodemographic, anthropometric, and environmental parameters for cases and controls.

	Cases	Controls	*p*-Value
	n = 61	n = 101	
Mean Age ± SD (years)	40.1 ± 13.8	36.3 ± 16.5	0.127
Female	32 (52.5)	62 (61.4)	0.265
Place of residence			0.965
Beirut	28 (45.9)	46 (45.5)	
Marital status	n = 59	n = 99	0.097
Single	29 (49.2)	62 (62.6)	
Married	30 (50.8)	37 (37.4)	
Academic level	n = 59	n = 99	0.230
Elementary	5 (8.5)	2 (2)	
High school graduate	5 (8.5)	7 (6.9)	
University	48 (81.4)	92 (91.1)	
Occupation	n = 57	n = 95	0.282
Liberal profession	15 (26.3)	18 (18.9)	
Employee	25 (43.9)	37 (38.9)	
Retired/Unemployment/Other	17 (29.8)	40 (42.1)	
Crowding index †	n = 50	n = 90	0.056
≤1	32 (64.0)	71 (78.9)	
>1	18 (36.0)	19 (21.1)	
Smoking (yes)	15 (24.6)	11 (10.9)	0.021
Other illnesses (yes)	n = 24	n = 18	0.676
Cardiovascular	7 (29.2)	7 (38.9)	
Metabolic	6 (9.8)	2 (11.1)	
Autoimmune	7 (11.5)	6 (33.3)	
Two or more of illnesses	4 (6.6)	3 (16.7)	
Family history of illnesses	n = 41	n = 70	0.378
Cardiovascular	7 (17.1)	17 (24.3)	
Metabolic	8 (19.5)	14 (20.0)	
Autoimmune	2 (4.9)	3 (4.3)	
Two or more of these	22 (53.7)	28 (40)	
Other	2 (4.9)	8 (11.4)	
Physically active (yes)	22 (36.1%)	52 (51.5%)	0.056

Continuous variables were reported as means ± standard deviations or median (interquartile range) when not normally distributed. Categorical variables were reported as numbers and percentages (n, (%)). Statistical tests used: independent *t*-test or Mann–Whitney U (continuous variables), χ^2^-test (categorical variables). SD = standard deviation, *p* < 0.05. † Crowding index: amount of residents/number of rooms. Some variables have missing data, and participant number (n) is included for reference. Patients were categorized as physically active according to the WHO recommendations, i.e., if they did at least 150 min of moderate-intensity aerobic physical activity or 75 min of vigorous-intensity aerobic physical activity.

**Table 2:** Flares, hospitalizations, and change in diet or medication over the previous year.

	UC	CD	*p*-Value	Total (n = 47)
	n = 25	n = 22		
Number of flares			0.939	
0	8 (32.0)	7 (31.8)		15 (31.9)
1	8 (32.0)	8 (36.4)		16 (34.0)
2 or more	9 (36.0)	7 (31.8)		16 (34.0)
Hospitalizations or surgeries			0.354	
Yes	5 (20.0)	7 (31.8)		12(25.5)
No	20 (80.0)	15 (68.2)		35 (74.5)
Change in IBD medications			0.995	
Yes	8 (32.0)	8 (36.4)		16 (34.0)
No	17 (68.0)	14 (63.6)		31 (66.0)
Change in diet due to flares			0.578	
Yes	14 (56.0)	15 (68.2)		29 (61.7)
No	11 (44.0)	7 (31.8)		18 (38.3)

Categorical variables were reported as numbers and percentages (n, (%)). Test used: χ^2^-test. UC = ulcerative colitis. CD = Crohn’s disease. IBD = inflammatory bowel disease.

**Table 3:** Energy, macronutrients, and micronutrients intake per day in patients with IBD versus controls.

	Cases (n = 61)	Controls (n = 101)	*p*-Value	DRI
Total Energy Intake (kcals)	1641 (1492–1997)	2546 (1994–3428)	0.001	1900–2900
Total Energy Intake (kcals) Among Men (n = 68)	1609 (1526–2136)	2588 (2174–3806)	0.001	2300–2900
Total Energy Intake (kcals/) Among Women (n = 94)	1656 (1477–1962)	2507 (1955–3288)	0.001	1900–2200
Protein (g)	77.5 (70.5–87.4)	99.5 (74.3–134.0)	0.001	52–56
Protein (g) Among Men	77.6 (68.2–93.4)	99.5 (76.6–136.5)	0.022	52–56
Protein (g) Among Women	76.9 (70.5–83.4)	98.8 (72.9–130.0)	0.001	46
Protein (%)	17.4 ± 3.2	16.5 ± 4.4	0.171	10–35
Lipids (g)	56.1 (49.1–77.7)	113.5 (84.9–148.0)	0.001	N/A
Lipids (%)	31.5 ± 6.0	37.6 ± 15.7	0.805	20–35
MUFAs (g)	21.1 (18.4–24.6)	36.0 (27.8–46.6)	0.001	N/A
MUFAs (%)	11.9 ± 2.6	12.8 ± 2.9	0.07	15–20
PUFAs (g)	9.7 (7.7–15.8)	18.4 (13.6–23.9)	0.001	N/A
PUFAs (%)	6.5 ± 2.9	6.4 ± 2.1	0.97	5–10
SFAs (g)	13.9 (9.4–22.7)	35.7 (26.0–48.1)	0.001	N/A
SFAs (%)	8.1 ± 3.4	12.4 ± 3.2	0.001	<10
Carbohydrates (g)	217.0 (196.5–262.6)	295 (218–369.8)	0.001	130
Carbohydrates (%)	50.1 ± 4.3	45.5 ± 5.6	0.001	45–65
Sucrose (g)	45.45 (35.8–74.7)	90.9 (67.3–1110)	0.001	50
Lactose (g)	5.7 (3.0–9.2)	9.6 (2.3–14.3)	0.036	N/A
Fiber (g)	18.2 (15.0–27.5)	29.1 (20.1–35.8)	0.001	21–38
Fiber (g) Among Men	18.6 (15.2–26.4)	31.2 (18.4–37.4)	0.007	30–38
Fiber (g) Among Women	17.7 (14.4–30.6)	28.9 (20.1–35.3)	0.002	21–26
Alcohol (yes), n (%)	10 (17.5)	22 (23.9)	0.358	N/A
Red Meat	n = 58	n = 92	0.024	N/A
0–3 times per month	16 (27.6)	46 (45.5)		
1–6 times per week	39 (67.2)	42 (45.7)		
At least once a day	3 (5.2)	4 (4.3)		
Dairy n, (%)	n = 58	n = 97	0.161	N/A
0–3 times per month	7 (12.1)	4 (4.1)		
1–6 times per week	32 (55.2)	55 (56.7)		
At least once a day	19 (32.8)	38 (39.2)		
Fruits	n = 58	n = 96	0.384	N/A
0–3 times per month	5 (8.6)	12 (12.5)		
1–6 times per week	23 (39.7)	45 (46.9)		
At least once a day	30 (51.7)	39 (40.6)		
Vegetables	n = 58	n = 94	0.836	N/A
Up to 6 times a week	28 (48.3)	47 (50)		
At least once a day	30 (51.7)	47 (50)		

Continuous variables were reported as means ± standard deviations or median (interquartile range) when not normally distributed. Categorical variables were reported as numbers and percentages. Statistical tests used: independent *t*-test or Mann–Whitney U (continuous variables), χ^2^-test (categorical variables). SD = standard deviation, *p* < 0.05. g = grams, kcal = kilocalories, MUFAs = mono-unsaturated fatty acids, PUFAs = poly-unsaturated fatty acids, SFAs = saturated fatty acids. N/A = not available. DRI: daily required intake [33].

## Data Availability

Data are not available in any repository but can be made available upon request.

## Supplementary Materials

The following supporting information can be downloaded at: https://www.mdpi.com/article/10.3390/nu16121826/s1, Table S1: Energy Intake, macronutrients, and micronutrients per day in patients with IBD, active versus remission; Table S2: Energy, macronutrients, and micronutrients per day in patients with IBD, according to Quality of life (QOL).
