# Supplementary material for: Nutritional Profile, Disease Severity, and Quality of Life of Patients with Inflammatory Bowel Disease: A Case–Control Study
Source: Nutrients. 2024 Jun 11;16(12):1826. doi: 10.3390/nu16121826 (PMC11206244; doi:10.3390/nu16121826)
Supplement: Supplementary file 1 [file nutrients-16-01826-s001.zip › nutrients-3031829-supplementary.pdf]

Supplementary appendix:

Contents

Supplementary Table S1: Energy intake, macronutrients, and micronutrients per day in patients with IBD, active versus re-mission. 2

Supplementary Table S2: Energy, macronutrients, and micronutrients per day in patients with IBD, according to Quality of life (QOL). 4

Supplementary Table S1. Energy intake, macronutrients, and micronutrients per day in patients with IBD, active versus re-mission.

	Active (n=19)	Remission (n=28)	p-value	DRI
**Total Energy intake (kcals)**	1612 (1521-1890)	1622 (1489-1851)	0.558	1900-2900
**Total Energy intake (kcals) among Men (n= 21)**	1605 (1547-1800)	1606 (1541-2190)	0.733	2300-2900
**Total Energy intake (kcals) among women (n= 26)**	1706 (1504-2610)	1644 (1400-1678)	0.153	1900-2200
**Protein (g)**	76.8 (70.3-82.7)	78.4 (73.4-88.8)	0.422	52-56
**Protein (g) Among Men (n=21)**	77.2 (70.3-82.7)	81.7 (76.2-94.7)	0.340	52-56
**Protein (g) Among Women (n=26)**	76.7 (68.5-84.2)	77.2 (72.0-81.6)	0.960	46
**Lipids (g)**	60.3 (53.9-71.3)	53.8 (46.8-65.7)	0.065	N/A
**MUFAs (g)**	20.3 (17.6-22.7)	21.1 (18.8-23.6)	0.308	N/A
**MUFAs (%)**	10.6 (9.8-12.8)	11.4 (11.1-12.6)	0.225	15-20
**PUFAs (g)**	11.7 (8.5-15.5)	5.6 (7.1-12.2)	0.041	N/A
**PUFAs (%)**	5.9 (4.7-7.7)	4.9 (4.3-6.1)	0.049	5-10
**SFAs (g)**	14.2 (10.0-17.6)	11.3 (9.2-15.5)	0.298	N/A
**SFAs (%)**	7.5 (5.8-8.6)	6.3 (5.4-8.2)	0.404	<10
**SFAs (g)**	14.2 (10.0-17.6)	11.3 (9.2-15.5)	0.298	N/A
**Omega-3 Fatty Acids (g)**	0.74 (0.5-1.2)	0.8 (0.5-1.4)	0.606	5-10%
**Omega-6 Fatty acids (g)**	6.5 (5.1-7.5)	5.0 (4.2-5.9)	0.012	0.6-1.2%
**Carbohydrates (g)**	218.5 (198.0-280.0)	213.5 (196.9-242.3)	0.441	130
**Sucrose (g)**	51.8 (34.7-70.8)	39.3 (34.9-51.5)	0.225	50
**Lactose (g)**	5.8 (3.5-100)	6.5 (3.7-9.7)	0.974	N/A

**Fibers (g)**	19.5 (14.9-33.3)	17.4 (15.2-21.9)	0.392	21-38
**Fibers (g) Among Men**	17.8 (14.6-21.9)	18.2 (15.5-2.2)	0.622	30-38
**Fibers (g) Among Women**	21.0 (14.8-39.6)	17.1 (14.1-20.0)	0.153	21-26
**Alcohol (yes), n (%)**	6 (31.6)	4 (14.3)	0.276	N/A
**Red Meats n, (%)**			0.083	N/A
**0-3 times per month**	5 (26.3)	7 (25.0)		
**1-6 times per week**	13 (68.4)	19 (67.9)		
**At least once a day**	1 (5.3)	2 (7.1)		
**Dairy n, (%)**			0.143	N/A
**0-3 times per month**	4 (21.1)	1 (3.6)		
**1-6 times per week**	9 (47.4)	14 (50.0)		
**At least once a day**	6 (31.6)	13 (46.4)		
**Fruits n, (%)**			0.030	N/A
**0-3 times per month**	0 (0)	4 (14.3)		
**1-6 times per week**	10 (52.6)	7 (25.0)		
**At least once a day**	9 (47.4)	17 (60.7)		
**Vegetables n, (%)**			0.859	N/A
**Up to 6 times a week**	10 (52.6)	14 (50.0)		
**At least once a day**	9 (47.4)	14 (50.0)		

Continuous variables were reported as means± standard deviations or Median (Interquartile range) when not normally distributed. Categorical variables were reported as numbers and percentages. Statistical tests used: independent t-test or Mann-Whitney U (continuous variables), χ^2-test (categorical variables). SD=standard deviation, $P<0.05$. g=grams, kcal=kilocalories, MUFAs= Mono-unsaturated fatty acids, PUFAs=poly-unsaturated fatty acids, SFAs=saturated fatty acids. N/A=Not available. DRI: Daily reference intakes.

Supplementary Table S2. Energy, macronutrients, and micronutrients per day in patients with IBD, according to Quality of life (QOL).

	LQOL (n=33)	HQOL (n=14)	p-value	DRI
**Total Energy intake (kcals)**	1688 (1566-2025)	1517 (1408-1644)	0.003	1900-2900
**Total Energy intake (kcals) Among Men (n= 21)**	1638 (1586-2190)	1514 (1450-1648)	0.058	2300-2900
**Total Energy intake (kcals) Among Women (n= 26)**	1697 (1511-1990)	1523 (1392-1645)	0.036	1900-2200
**Protein (g)**	81.4 (72.3-89.2)	75.1 (69.4-77.9)	0.019	52-56
**Protein (g) Among Men (n=21)**	81.7 (77.0-94.7)	76.5 (65.8-79.0)	0.095	52-56
**Protein (g) Among Women (n=26)**	81.2 (69.6-85.9)	73.6 (70.4-78.6)	0.115	46
**Lipids (g)**	60.3 (53.8-74.4)	49.9 (42.3-56.7)	0.002	N/A
**MUFAs (g)**	21.2 (18.6-24.8)	20.5 (18.4-21.6)	0.301	N/A
**MUFAs (%)**	11.2 (10.3-12.7)	11.6 (11.1-12.9)	0.323	15-20
**PUFAS (g)**	11.7 (7.8-15.3)	8.2 (6.8-9.2)	0.011	N/A
**PUFAs (%)**	6.0 (4.5-7.5)	4.8 (4.6-5.2)	0.034	5-10
**SFAs (g)**	14.7 (10.9-22.5)	10.0 (6.9-11.1)	0.001	N/A
**SFAs (%)**	7.6 (6.1-9.7)	6.0 (4.3-6.4)	0.002	<10
**Omega-3 Fatty Acids**	0.7 (0.5-1.2)	1.1 (0.4-1.1)	0.454	N/A
**Omega-6 Fatty acids**	5.4 (4.9-6.9)	4.9 (3.7-6.5)	0.081	5-10%
**Carbohydrates (g)**	218.5 (200.0-270.3)	207.8 (186.8-218.1)	0.034	0.6-1.2 %
**Sucrose (g)**	51.0 (36.8-75.1)	37.1 (33.0-41.2)	0.016	130
**Lactose (g)**	6.4 (3.5-11.3)	6.2 (3.8-8.7)	0.745	50
**Fibers (g)**	18.2 (15.1-25.7)	16.1 (14.6-21.4)	0.226	N/A

**Fibers (g) Among Men (n=21)**	18.2 (15.2-24.1)	17.7 (15.5-21.4)	0.970	21-38
**Fibers (g) Among Women (n=26)**	17.9 (14.9-34.4)	15.1 (14.0-21.0)	0.238	30-38
**Alcohol (yes), n (%)**	8 (24.2)	2 (14.3)	0.591	21-26
				N/A
				N/A
				N/A
				N/A
				N/A

Continuous variables were reported as means± standard deviations or Median (Interquartile range) when not normally distributed. Categorical variables were reported as numbers and percentages. Statistical tests used: independent t-test or Mann-Whitney U (continuous variables), $\chi 2$-test (categorical variables). SD=standard deviation, $P<0.05$. g=grams, kcal=kilocalories, MUFAs= Mono-unsaturated fatty acids, PUFAs=poly-unsaturated fatty acids, SFAs=saturated fatty acids. N/A=Not available. Kcal: Kilocalories. DRI: Daily reference intakes.
